# The what and how of video analysis research in rugby union: a critical review

**DOI:** 10.1186/s40798-018-0142-3

**Published:** 2018-06-18

**Authors:** Steve den Hollander, Ben Jones, Michael Lambert, Sharief Hendricks

**Affiliations:** 1 Division of Exercise Science and Sports Medicine, Department of Human Biology, Faculty of Health Sciences, University of Cape Town and Sports Science Institute of South Africa, Cape Town, South Africa; 2 Institute for Sport, Physical Activity and Leisure, Leeds Beckett University, Leeds, UK; 3 Yorkshire Carnegie Rugby Union Football Club, Leeds, UK; 4 Leeds Rhinos Rugby League Club, Leeds, UK; 5 The Rugby Football League, Leeds, UK

**Keywords:** Video analysis, Critical review, Rugby

## Abstract

**Background:**

Video analysis is a common tool used in rugby union research to describe match performance. Studies using video analysis range from broad statistical studies of commercial databases to in-depth case-studies of specific match events. The range of types of studies using video analysis in rugby union, and how different studies apply the methodology, can make it difficult to compare the results of studies and translate the findings to a real-world setting. In attempt to consolidate the information on video analysis in rugby, a critical review of the literature was performed.

**Main body:**

Ninety-two studies were identified. The studies were categorised based on the outcome of the study and the type of research question, sub-categorised as ‘what’ and ‘how’ studies. Each study was reviewed using a number of questions related to the application of video analysis in research.

There was a large range in the sample sizes of the studies reviewed, with some of the studies being under-powered. Concerns were raised of the generalisability of some of the samples. One hundred percent of ‘how’ studies included at least one contextual variables in their analyses, with 86% of ‘how’ studies including two or more contextual variables. These findings show that the majority of studies describing how events occur in matches attempted to provide context to their findings. The majority of studies (93%) provided practical applications for their findings.

**Conclusion:**

The review raised concerns about the usefulness of the some of the findings to coaches and practitioners. To facilitate the transfer and adoption of research findings into practice, the authors recommend that the results of ‘what’ studies inform the research questions of ‘how’ studies, and the findings of ‘how’ studies provide the practical applications for coaches and practitioners.

**Electronic supplementary material:**

The online version of this article (10.1186/s40798-018-0142-3) contains supplementary material, which is available to authorized users.

## Key points

- Sample size calculations should be adopted in video analysis research.
- A consensus is needed for the definition and use of variables in video analysis research of rugby union.
- To facilitate the transfer and adoption of research findings into practice, a sequence of applied video analysis research should be adopted

## Background

Rugby union is a high-intensity collision based sport [1]. It is played by over 6.6 million players, across 199 countries, which makes it one of the most played sports in world [2]. The sport generated £385 million revenue in 2015 and winning major international competitions is the ultimate goal for national teams [3]. Rugby union is also associated with a higher risk of injury, compared to other sports like Association Football [4]. The higher injury risk is due to the dynamic environment in which physical contact occurs between players, with the tackle accounting for more than 50% of all match-related injuries [5].

The drive to reduce the risk of injury and improve performance in rugby has set in motion a high volume of scientific research including the analysis of match video footage to identify and describe player and team actions [6, 7], usually in relation to performance or injury outcomes [8]. Arguably, a strength of video analysis is that it allows for dynamic and complex situations in sports to be quantified in an objective, reliable and valid manner [9].

Video analysis research in rugby union frequently includes *what* studies that identify key events (for example, number of tackles in a match) to *how* studies that describe key events (for example, tackle technique relates to injury). Furthermore, the scope of these studies range from the description of in-depth case studies [10–12] to the broad analysis of commercial data bases [13–15]; and from studies that apply sophisticated statistical modelling that accounts for context [16–18] to studies that only report on the frequencies of events [19–21]. The sizes and types of samples used in these studies also vary considerably, a similar finding to that in Association Football (for a review: see Mackenzie and Cushion, 2013 [22]).

Due to the many different types of studies using video analyses in rugby, it is difficult to standardise the techniques. This makes it difficult to compare studies and translate the findings to a real-world setting. In response to this, a critical review of the literature on video analysis research in rugby union was performed. The aim was to critically appraise the studies to determine how the findings can be used to inform practise.

## Main text

### Methods

The purpose of a critical review is to show an extensive overview of the literature, as well as a critical evaluation of the quality of the literature [23]. It exceeds a narrative review of the studies by including a degree of analyses [23]. The methods of a systematic review were used in the literature search [24, 25]. This was done to ensure that all the available relevant literature were included in the review [23]. The critical evaluation of the literature was performed through the use of a series of polar questions (Table 1). In line with the purpose of the review, these questions were related to the methodology of the studies, namely, how the researchers used video analysis methods to collect data and answer specific research questions. Polar questions were used to attempt to provide a level of objectivity to the evaluation.

**Table 1:** Polar questions used to review literature

Sample type	
Was a complete season/tournament analysed?	
Was the research from a one-off tournament (example, World Cup)?	
Did the research include data from multiple seasons or tournaments?	
Were differentiations made between competition stages?	
Operational definitions	
Were the variables analysed fully defined?	
Were the variables partially defined?	
Was reference made to a previous publication, or the development of definitions, but not provided in the article?	
Were definitions provided insufficient?	
Match-related context	
Was the relative strength of the opposition considered in the analysis?	
Was there a reference made to the match location?	
Were environmental factors taken into account? (Weather, field condition)	
Event-related context	
Was there a comparison between different outcomes?	
Was the playing position included in the analysis and differentiated in the results?	
Was the field position taken into consideration?	
Was there specific information relating to the playing situation of the assessed variables? (Formation or movement of the attacking and defensive lines, the number of support players, the type of pass/kick, etc.)	
Was technique assessed? (injury studies only)	
Practical application	
Was there a reference to the practical application of the findings?	

#### Systematic literature search

Specific search terms were used to identify peer-reviewed articles in three electronic databases, SCOPUS, PubMed and Web of Science. The search terms were ‘rugby union’ in the title, keywords or abstract linked with either ‘performance analysis’, ‘video analysis’, ‘tackle performance’, ‘video’, ‘notational analysis’, ‘match performance’, ‘match analysis’, ‘time motion analysis’, ‘attacking strategies’, ‘defensive strategies’, ‘performance indicators’, ‘injury risk’, ‘injury mechanisms’ or ‘injury rates’ anywhere in the text. The time frame for the literature search was any study published before 2017. The search results from the three databases were merged, and any duplicates were removed.

The inclusion criteria were as follows: the article needed to use video analysis to quantitatively study rugby union match footage and needed to be published, in English, in a peer-reviewed journal. Inclusion criteria were applied at the title, abstract and full-text level, and any article not meeting the criteria was omitted from the review. Inter-rater reliability testing was conducted for this process of the literature search. A second author applied the inclusion criteria to the merged database at the title, abstract and full-text level. Where there were any disparities between the two databases, the reasons for including or excluding the relevant papers were discussed and the studies were either included or excluded from the final database.

**Fig. 1:**
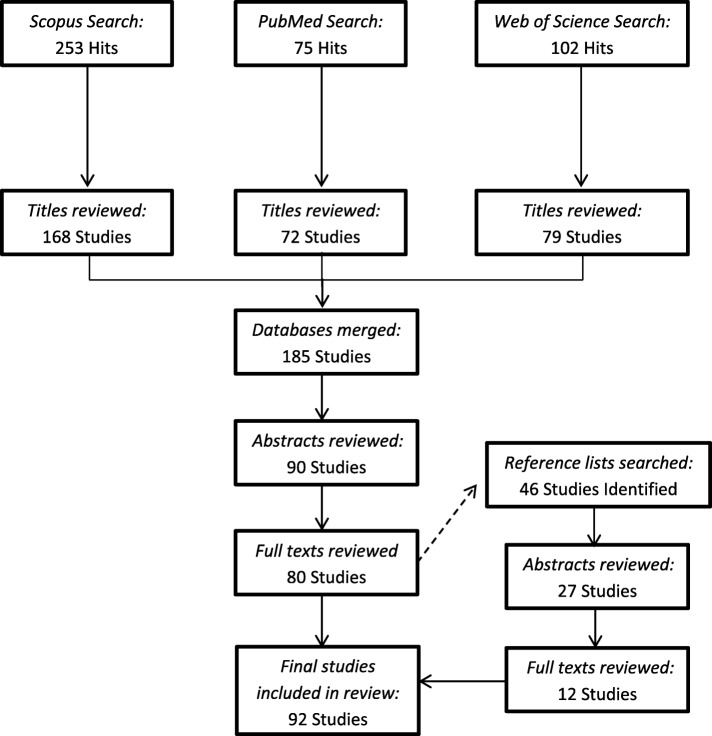
Flow diagram of literature search

The reference lists of the papers that met the inclusion criteria were checked, and any relevant papers were added to a separate database. Inclusion criteria were applied to this database, at abstract and full-text levels. The papers that met the criteria were merged into the original database. The outcome of this process was a total of 92 papers (Fig. 1).

#### Critical evaluation

Data related to the aims, outcomes, variables investigated, sample sizes and type, and key findings of the studies were extracted from the identified papers. The identified papers were categorised into three groups based on the outcomes of the paper; *physical demands*, *performance* and *injury*. Seventeen studies did not fall under these groups and were reviewed under the category *other*. Within these categories, the studies were further categorised into ‘what’ and ‘how’ studies, based on the research question. Studies that identified the frequencies of specific variables were categorised as what studies. These were typically studies which used broad statistical analyses of large databases. Studies that identified the associations between different variables to describe how an event occurred were categorised as how studies. Grouping the studies into these two categories allowed for more homogenous comparisons during the review process.

**Fig. 2:**
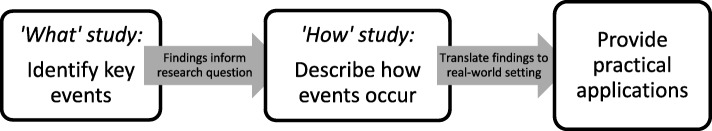
The sequence of applied video analysis research of match performance

Furthermore, classifying the studies into these two groups also allowed for different requirements for the different types of video analysis studies. Video analysis research involves the analysis of the frequencies or counts of specific variables, termed key performance indicators (KPI) [26]. Typically, ‘what’ studies identify KPIs associated with specific outcome. The primary requirement for ‘what’ studies is that the samples used are sufficiently large so that the findings are generalisable. It is also important that the samples are representative of the general rugby population, including multiple teams, seasons or levels of play, for the findings to be considered useful. The crucial requirement for ‘how’ studies are that contextual variables are included in their analyses. The purpose of these studies is to understand how an outcome occurs. As rugby is a dynamic sport, any finding must provide or account for the context in which the finding occurred for it to be applicable [27]. This brings up the final requisite for the studies. With the view that video analysis research should be progressive, the research questions of *how* studies should be based on the findings of *what* studies, and the practical applications of the research, based on the findings of *how* studies (Fig. 2).

With these requirements in mind, a number of polar questions (Table 1) were developed to review the studies. The questions were developed through the use of previous literature [22], and questions developed specifically for this review. The questions specifically addressed areas of criticism of performance analysis research [8, 22, 27]. The first set of questions evaluated the sample selected for the study, and the second the provision of definitions for the variables used in the analysis. The third group of questions evaluated the inclusion of variables that provide context to the event analysed. A common criticism of video analysis is that it has a tendency towards reductionism [8, 28, 29]. If the actions identified and described in these studies are analysed in isolation, the context in which they occur can be lost. A number of approaches have been suggested on how to provide context [8, 27, 29, 30], which all involve identifying patterns between the event identified in the study and specific task and environmental variables (contextual variables) related to the analysed event or match. The questions used in this review evaluated the number of contextual variables included in studies. The final question identified whether or not the studies provided practical applications for their findings.

#### Statistical analysis

The results of the critical evaluation were analysed using descriptive statistics, to describe and compare the frequency of occurrences.

### Results

**Fig. 3:**
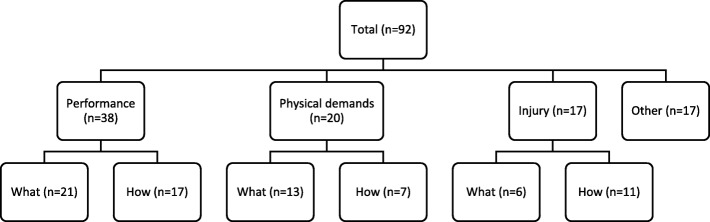
Categories of video analysis studies; *n* = the number of studies

A total of 92 studies were included in the review. The papers were categorised into three groups (i.e., performance, physical demands, injury) based on the outcomes of the paper (Fig. 3). Seventeen papers did not fall into these categories; the outcomes of these papers included the development and comparisons of tools [31–36], touchline safety [37], decision-making behaviours [38], and the effects of law changes [39–43], professionalism [44–46], and time [47] on various match characteristics.

#### Sample size and selection

Three out of 21 performance-related studies in the sub-category ‘what’ had sample sizes larger than 100 games. Forty-seven percent of these studies included data from multiple competitions or seasons, and 38% of the samples were from one-off tournaments that do not occur annually. Tables 2 and 3 provide a summary of the sample sizes and types used in the studies.

**Table 2:** A summary of the sample sizes

Sample size	Physical demands				Performance				Injury related			
	What		How		What		How		What		How	
	Yes (*n*)	Studies	Yes (*n*)	Studies	Yes (*n*)	Studies	Yes (*n*)	Studies	Yes (*n*)	Studies	Yes (*n*)	Studies
Number of matches												
< 10	8	[1, 49, 50, 78–82]	3	[83–85]	4	[56, 57, 86, 87]	5	[11, 12, 72, 76, 88]	1	[89]	0	
10–35	2	[90–92]	3	[71, 93, 94]	8	[20, 58]	8	[16, 17, 19, 65, 101–104]	1	[105]	1	[61]
36–100	2	[106, 107]	0		6	[15, 18, 21, 55, 75, 108]	2	[62, 109]	1	[110]	1	[63]
101–200	0		0		0		2	[53, 54]	0		0	
201–300	0		0		1	[13]	0		1	[111]	0	
300+	0		0		2	[14, 51]	0		2	[5, 52]	0	
Not published	0		1	[68]	0		0					
Number of players												
< 10	0		0		0		0		0		0	
11–20	2	[78, 79]	0		0		0		0		0	
21–30	5	[3, 4, 7, 22, 24]	2	[68, 83]	0		0		1	[89]	0	
31–40	1	[91]	1	[85]	0		0		0		0	
41–50	0		1	[93]	0		0		0		0	
51–100	0		1	[94]	0		0		0		0	
101–200	0		0		0		0		0		0	
201–300	0		1	[71]	0		0		0		0	
300+	2	[106, 107]	0		1	[13]	0		0		0	
Not published	1	[82]	0		0		0		0		0	
Number of events												
< 20	0				0		0		0		2	[10, 70]
21–30	0				0		2	[11, 12]	0		1	[60]
31–40	0				1	[57]	0		0		0	
41–50	0				0		1	[19]	0		1	[112]
51–100	2	[50, 81]	1	[84]	0		0		0		4	[69, 73, 74, 113]
101–200	0				1	[20]	0		0		0	
201–300	0				0		0		0		0	
301–400	0				0		3	[54, 88, 104]	1	[105]	1	[114]
401–500	0				1	[58]	0		0		0	
501–1000	0				1	[96]	2	[103, 109]	0		0	
1001–2500	0				0		5	[16, 20, 36, 40, 41]	0		0	
2501–5000	0				0		0		0		0	
5000+	0				2	[15, 21]	1	[62]	3	[57, 76, 84]	2	[61, 63]

**Table 3:** A summary of the types of samples selected

Sample	Yes (*n*)	Studies	No (*n*)	Studies	N/A (*n*)	Studies
Physical demands—what						
Complete season/tournament?	2	[106, 107]	11	[1, 50, 78–82, 90–92]		
Is the research from a one-off tournament(s)?	2	[81, 107]	11	[1, 50, 78–80, 82, 90–92, 106]		
Includes data from more than one season/tournament?	4	[78–80, 106]	9	[1, 49, 50, 81, 82, 90–92, 107]		
Did the study differentiate between competition stages?	2	[50, 81]	10	[1, 49, 78–80, 82, 90, 92, 106, 107]	1	[91]
Physical demands —how						
Complete season/tournament?	0		7	[68, 71, 83–85, 93, 94]		
Is the research from a one-off tournament(s)?	0		7	[68, 71, 83–85, 93, 94]		
Includes data from more than one season/tournament?	5	[68, 71, 84, 93, 94]	2	[83, 85]		
Did the study differentiate between competition stages?	0		5	[71, 83, 84, 93, 94]	2	[68, 85]
Performance—what						
Complete season/tournament?	12	[13–15, 18, 20, 21, 51, 55, 57, 75, 97, 108]	9	[56, 58, 86, 87, 95, 96, 98–100]		
Is the research from a one-off tournament(s)?	8	[55–57, 87, 95, 97, 99, 100]	13	[13–15, 18, 20, 21, 51, 58, 75, 86, 96, 98, 108]		
Includes data from more than one season/tournament?	11	[13–15, 18, 20, 21, 51, 58, 75, 96, 108]	10	[55–57, 86, 87, 95, 97–100]		
Did the study differentiate between competition stages?	4	[21, 87, 99, 100]	15	[13–15, 20, 51, 55–58, 75, 86, 95–98]	2	[18, 108]
Performance—how						
Complete season/tournament?	5	[53, 54, 65, 102, 109]	12	[11, 12, 16, 17, 19, 62, 72, 76, 88, 101, 103, 104]		
Is the research from a one-off tournament(s)?	2	[88, 109]	15	[11, 12, 16, 17, 19, 53, 54, 62, 65, 72, 76, 101–104]		
Includes data from more than one season/tournament?	2	[101, 104]	15	[11, 12, 16, 17, 19, 53, 54, 62, 65, 72, 76, 88, 102, 103, 109]		
Did the study differentiate between competition stages?	0		15	[12, 16, 17, 53, 54, 62, 65, 72, 76, 88, 101–104, 109]	2	[11, 19]
Sample (cont.)	Yes (*n*)	Studies	No (*n*)	Studies	N/A (*n*)	Studies
Injury—what						
Complete season/tournament?	3	[5, 52, 111]	3	[89, 105, 110]		
Is the research from a one-off tournament(s)?	0		6	[5, 52, 89, 105, 110, 111]		
Include data from more than one season/tournament?	4	[5, 52, 105, 110]	2	[89, 111]		
Did the study differentiate between competition stages?	0		1	[5]	5	[52, 89, 105, 110, 111]
Injury—how						
Complete season/tournament?	3	[74, 113, 114]	8	[10, 60, 61, 63, 69, 70, 73, 112]		
Is the research from a one-off tournament(s)?	0		11	[10, 60, 61, 63, 69, 70, 73, 74, 112–114]		
Include data from more than one season/tournament?	10	[10, 60, 61, 63, 69, 73, 74, 112–114]	1	[70]		
Did the study differentiate between competition stages?	0		4	[10, 70, 73, 112]	7	[60, 61, 63, 69, 74, 113, 114]

Not applicable

#### Definitions of variables

Fifty percent of the studies provided full definitions for the variables used in the analyses. In 19% of the studies, the variables were partially defined, 5% made reference to definitions published elsewhere and 26% provided insufficient definitions. A summary of the operational definitions provided can be found in Table 4.

**Table 4:** A summary of the definitions provided for all studies

Definitions provided	Number of studies (*n*)	Percentage of total (%)
Fully defined	46	50.0
Partially defined	17	18.5
Reference made to definition	5	5.4
Insufficiently defined	24	26.1

#### Context

Less than half of the sub-category ‘how’ studies included match-related contextual variables in their analyses (16 out of 35). Twenty-six percent of the studies included variables related to the opposition strength, 8% variables related to match location and 6% of studies included variables related to environmental conditions.

Nineteen out of 35 sub-category ‘how’ studies (54%) included more than three event-related contextual variables in their analysis. Eighty-four percent of performance related studies and 64% of injury studies included variables related to the outcome of the event. One hundred percent of studies in the category physical demands included and differentiated between variables related to playing position, compared to 47% of performance studies and 45% of injury studies. Seventy-three percent of injury-related studies and 59% of performance studies included variables which describe the playing situation. A summary of the use of contextual variables can be found in Tables 5 and 6.

**Table 5:** The number of categories of contextual variables included in the analysis; where a category was not applicable to the study, it was counted as included

Number of matched categories included	Number of studies (*n*)	Studies
0	19	[10–12, 16, 17, 19, 60–62, 65, 71, 76, 84, 93, 94, 101, 104, 109, 112]
1	13	[53, 54, 63, 69, 70, 72, 73, 83, 85, 88, 102, 113, 114]
2	3	[68, 74, 103]
3	0	
Number of event categories included	Number of studies (*n*)	Studies
0	1	[70]
1	7	[10, 53, 60, 65, 104, 113, 114]
2	8	[11, 12, 62, 63, 72, 85, 88, 112]
3	16	[16, 17, 19, 61, 68, 71, 73, 74, 76, 83, 84, 93, 94, 101, 102, 109]
4	3	[54, 69, 103]

**Table 6:** A Summary of the ‘how’ studies that included contextual variables in the analyses

Context	Yes (*n*)	Studies	No (*n*)	Studies	N/A (*n*)	Studies
Physical demands						
Was the strength of the opposition considered?	1	[85]	6	[68, 71, 83, 84, 93, 94]		
Was the match location considered?	0		5	[71, 84, 85, 93, 94]	2	[68, 83]
Were environmental factors considered?	1	[68]	6	[71, 83–85, 93, 94]		
Was there a comparison between outcomes?	3	[71, 84, 85]	4	[68, 83, 93, 94]		
Were the playing positions considered?	7	[68, 71, 83–85, 93, 94]	0			
Performance						
Was the strength of the opposition considered?	4	[54, 72, 102, 103]	13	[11, 12, 16, 17, 19, 53, 62, 65, 76, 88, 101, 104, 109]		
Was the match location considered?	2	[53, 103]	14	[11, 12, 16, 17, 19, 54, 62, 65, 72, 76, 101, 102, 104, 109]	1	[88]
Were environmental factors considered?	0		17	[11, 12, 16, 17, 19, 53, 54, 62, 65, 72, 76, 88, 101–104, 109]		
Was there a comparison between outcomes?	14	[12, 16, 17, 19, 53, 54, 62, 65, 72, 76, 88, 101–103]	3	[11, 104, 109]		
Were the playing positions considered?	7	[16, 19, 54, 76, 101, 103, 104]	8	[12, 17, 53, 62, 65, 72, 88, 102]	2	[11, 109]
Was the field location of the events considered?	9	[11, 12, 17, 19, 54, 101–103, 109]	8	[16, 53, 62, 65, 72, 76, 88, 104]		
Was there specific information relating to the playing situation of the assessed variables?	10	[16, 17, 54, 62, 72, 76, 88, 102, 103, 109]	7	[11, 12, 19, 53, 65, 101, 104]		
Injury						
Was the strength of the opposition considered?	0		11	[10, 60, 61, 63, 69, 70, 73, 74, 112–114]		
Were environmental factors considered?	1	[69]	10	[10, 60, 61, 63, 70, 73, 74, 112–114]		
Was there a comparison between outcomes?	7	[61, 63, 69, 73, 74, 113, 114]	4	[10, 60, 70, 112]		
Were the playing positions considered?	5	[61, 69, 73, 74, 112]	6	[10, 60, 63, 70, 113, 114]		
Was there specific information relating to the playing situation of the assessed variables?	7	[10, 60, 61, 63, 69, 73, 74]	4	[70, 112–114]		
Was technique assessed?	7	[60, 63, 69, 74, 112–114]	4	[10, 61, 70, 73]		

Not applicable

#### Practical application of studies

Eighty-one percent of studies identified in this review provided practical applications for their findings. Differentiating between ‘what’ and ‘how’ studies showed that 76% of ‘what’ studies provided practical applications compared to 86% of ‘how’ studies. Table 7 provides a summary of these results.

**Table 7:** A summary of the reference to practical application

Reference to practical application		Yes (*n*)	Studies	No (*n*)	Studies
Physical demands	What	12	[1, 49, 50, 78–82, 90–92, 106, 107]	0	
	How	6	[68, 71, 83, 85, 93, 94]	1	[84]
Performance	What	13	[13–15, 18, 20, 55, 75, 87, 96–98, 100, 108]	8	[21, 51, 56–58, 86, 95, 99]
	How	13	[11, 16, 17, 19, 53, 54, 62, 65, 72, 101–103, 109]	4	[12, 76, 88, 104]
Injury	What	5	[5, 52, 89, 105, 111]	1	[110]
	How	11	[10, 60, 61, 63, 69, 70, 73, 74, 112–114]	0	

### Discussion

The video analysis of match footage is a common tool used to provide researchers with objective, quantifiable data about match performance [7]. Although video analysis studies are often grouped together, there is a large disparity in the type of data gathered and the level of analysis conducted within these studies. The studies range from broad statistical analyses of commercial databases to more in-depth case studies [48]. As a result of this disparity, the findings of these studies have been challenged because of the questionable generalisability of the findings, and the reductionist nature of some of the analyses [22, 27, 29, 30]. In response to this a critical review of video analysis research in rugby union was performed, appraising the samples used, the provision of definitions to the variables analysed, the inclusion of contextual variables in the analysis and the provision of practical applications for the findings.

#### Sample size and selection

There was a large range in the sample sizes of the studies identified in this review. Sample sizes range from three studies with samples of less than five matches [11, 49, 50], to four studies analysing over 300 matches [5, 14, 51, 52]. Two of the studies with samples of less than 5 matches [49, 50] were not purely video analysis studies and involved taking blood samples of the players. This may account for the small samples. The other study, a case study [11], was categorised as a ‘how’ study and required the analyst to code each match manually. The four studies with large samples were all categorised as ‘what’ studies and had access to large commercial or team databases for their analyses. However, differentiating the studies into ‘what’ and ‘how’ studies did not drastically reduce the range in sample size. Within the sub-category ‘what’, 13 studies had samples of less than 10 games, in contrast to the four studies with samples of over 300 games. Similarly, within the ‘how’ sub-category, samples ranged from one study with a sample of 35 min of four games [49] to two studies which analysed 125 matches [53, 54]. There is, therefore, a need for a consensus on the sample size that would accurately reflect the rugby union population.

Not all studies described the samples used in terms of the number of matches analysed. Some studies described their samples in terms of the number of players investigated, and some by the number of events analysed (Table 2). Interestingly, there was an association between the three outcome categories of studies identified in this review and the description of the sample. For example, ‘physical demands’ studies predominantly describe their samples in terms of *players* analysed, whereas ‘performance’ studies refer to the number of *matches* analysed, and ‘how’ ‘performance’ studies focus largely on the number of events. The ‘injury’ studies described *matches* in the sub-category of ‘what’ studies and *events* in the ‘how’ sub-category of studies. This suggests that any consensus statement would need to differentiate between the different categories and/or sub-categories.

A requisite of ‘what’ studies is that the samples are sufficiently large to allow for general claims to be made from their results. In the context of 129 games in an English Premiership season, or 135 in a Super Rugby season, only 3 of the 21 performance studies (14%) and 3 of the 6 injury studies (50%) investigated 100 matches or more. One third of the performance studies specifically analysed matches from the Rugby World Cup, a competition that only consists of 48 matches. Only one of these studies [55] analysed all 48 matches, in comparison with two studies with samples of five matches [56, 57]. Furthermore, the effect of the change of time [44–47] and competition [58, 59] on match characteristics questions the validity of analysing one-off tournaments and highlights the importance of including multiple seasons or competitions in samples to improve the generalisability of the results. However, 10 out of 21 performance studies included only one season or competition in their sample, and 8 studies were from one-off tournaments. These findings question the generalisability of the samples, and subsequently the results. The results from the injury-related ‘what’ studies are more positive, with 67% of studies including data from multiple seasons or competitions, and none of the studies analysing one-off tournaments.

In ‘how’ studies, it was more applicable to refer to the number of events analysed, than matches. Although all 17 studies in this sub-category reported the number of matches analysed, with the exception of George et al. (2015) [53], the studies did not analyse entire matches; instead they analysed certain events and outcomes identified in matches which were specific to the aims of the particular study. There is a large range in the number of events analysed in these studies, with some studies reporting samples of 20–30 events [11, 12, 60], and others with more than 5000 events [61–63]. However, as the frequency of different events differs within matches, the statistical power of a sample cannot simply be assessed by the number of events analysed. For example, at first glance, a study of 8653 events [62] would seem to have more statistical power than a study of 362 events [54]. The first study analysed rucks and the second line breaks. In a match, there are approximately 142 rucks [62], compared to an average of three line breaks per match [54]. The line breaks study, thus, coded 125 matches to identify and analyse the 362 line break events [54]. The study that analysed rucks, analysed 8563 rucks in 60 matches [62]. Therefore, although the one study analysed far fewer events than the other, it analysed more than twice as many matches. This provides a challenge when assessing the individual merits of each study. Reporting sample size calculations may provide a more suitable basis to evaluate sample sizes [22]. Unfortunately, only one of the 35 sub-category ‘how’ studies identified in this review reported a sample size calculation [61].

Studies in the category physical demands aim to identify and describe the physical demands of playing a rugby union match. A study of the match-to-match variability of high-speed activities in football [64] showed that a sample size of at least 80 players would have sufficient statistical power to make meaningful inferences about the physical demands of match play. If that number is taken as a sufficiently powered sample, only three ‘physical demands’ studies had samples larger than 80 players. This suggests that 76% of the studies were underpowered.

#### Definitions of variables

There was a lack of clarity and transparency in the definitions of the variables used in the studies. Only 50% of studies fully defined the variables used in their analysis, with 26% providing no definitions. As a result, it becomes difficult for other researchers to compare the results of these studies or replicate them [22]. What further compounds this problem is that definitions of variables differ. For example, one study [65] used the International Rugby Board’s definition of a tackle, where a ball carrier needs to be brought to ground for a tackle to occur [66], whereas other studies have defined a tackle as any attempt to stop or impede a ball carrier, whether or not the ball carrier is brought to ground [5, 61]. Although both studies are analysing tackles, they may not always be analysing the same event. Therefore, comparisons between the findings of these studies need to be interpreted with caution. This review highlights the need for a consensus among researchers using video analysis in rugby union, on the operational definitions of variables used in rugby research.

#### Context

Particularly in ‘how’ studies, it is important that the frequency of KPIs are not analysed in isolation, but that the context in which the KPI occurs is included in the analysis. A number of approaches have been suggested on how to provide context to the KPIs; through the use of ecological system dynamics [8, 27], through a constraints-based approach [29] or through temporal pattern analyses [7]. All of these approaches involve identifying patterns between the identified KPIs and specific task and environmental variables (contextual variables) related to the analysed event or match.

The first group of variables provide context to the match that was analysed. The relative strength of the opposition, the location of the match or the environmental conditions may alter a team’s tactics and, therefore, have an effect on the frequency of a KPI [54, 67]. In an analysis of line breaks, den Hollander and colleagues found that teams created more line breaks when playing against weaker opposition, compared to equally ranked or stronger opposition [54]. Similarly, George and colleagues (2015) found that teams created more line breaks, missed fewer tackles and scored more points playing at home, compared to playing away [53]. Yet, only 9 out of 35 of the studies (26%) accounted for opposition strength, 8% differentiated between match location, and only 2 studies (6%), (1 study on physical demands [68] and 1 injury study [69]) included environmental conditions in their analysis. Information regarding environmental conditions, like rainfall, can be difficult to gather retrospectively. Weather websites usually provide information about the amount of precipitation there was on the day of the match, but not the specific time or consistency of the rainfall. Overall, the inclusion of variables that give context to the match was poor. Over half the studies reviewed did not include any match-related variables in their analysis, and only three studies included two of the three categories of match variables in their analyses.

The results of studies that included variables that provide context to the event analysed were more positive. The majority of studies included more than three out of a possible four categories and only one study did not include any contextual variables [70]. The category of context included seemed to depend on the type of study. The majority of performance studies included the match or event *outcome* in their analysis, most of injury studies included variables which described the *playing situation* in their analysis, and every physical demands study included *playing position* in their analysis.

To be useful, KPIs need to relate to an outcome [30]. For example, comparing the frequencies of KPIs with successful and unsuccessful events, injury and non-injury events or different outcomes to a phase of play enables the researcher to determine if a variable is specifically related to the event or if it occurs in general. In this way, one outcome acts as a control for another outcome which also allows researchers to apply more sophisticated probability statistics [54]. The comparison of outcomes was common in both performance (84%) and injury (64%) studies. The inclusion of outcome variables was less common in physical demands studies. Only three of the seven studies compared match or event outcomes, and only one of those studies was related to the distances players cover in a match. Interestingly, this study found no differences in the physical movement patterns between winning and losing teams [71].

There are clear physiological differences in the match demands between forwards and backline players in rugby union [67], and therefore it is not surprising that 100% of the physical demands studies differentiated between playing positions. Studies have also shown differences in skill demands between playing positions [15, 19, 54]. Van Rooyen (2012) reported differences between the number of tackles made by forwards and backs, with back row forwards attempting and completing more tackles than any other positional group [15]. Positional differences have also been found in the number of line breaks made, with backline players more likely to complete line breaks, compared to forwards [19, 54], and significant differences in the types of skills used by inside and outside backs in the build-up play leading to line breaks [54]. Despite these findings highlighting the difference in skill demands between positions, only 47% of performance studies and 45% of injury studies differentiated between playing position.

The category playing situation accounts for variables that describe the situation in which the event occurred. These can be variables that describe the interactions between teammates and opposition players. Examples of this are studies that analysed the interactions between attacking and defensive line shapes and movements when identifying key variables [17, 54, 62, 72]. Similarly, some studies analysed the interactions between opposing players in contact [16, 60, 61, 73, 74]. As this category was specific to events, and physical demands studies mainly described the demands of entire matches and not events, only studies related to performance and injuries were reviewed in this category. Most of the studies reviewed attempted to account for the playing situation, with 73% of injury studies and 59% of performance studies including variables related to the playing situation.

These findings show that most of the ‘how’ studies reviewed attempted to provide context for their results, although perhaps more attention could be given to variables related to the match context. The authors also acknowledge there are restrictions and limitations in including too many variables in an analysis. Many journals have word count restrictions, which impacts on the number of variables a study can report on. A study may, thus, have initially included variables in their analysis, but not included them in the publication as the findings were insignificant. Authors may also divide their study up into multiple papers, and unless read together the context of their findings may be lost. Despite these limitations, all of the ‘how’ studies reviewed included at least one contextual variable in their analyses, and 30 of the 35 papers included at least two types of contextual variables in their analyses.

#### Practical application of studies

A primary purpose of video analysis is to provide individuals involved in sports with objective and reliable information which can be used to inform practice [26]. Therefore, it is not surprising that 93% of studies gave practical applications for their findings. However, it is debatable whether all these findings, specifically those from ‘what’ studies, provide practical information [22]. For example, a study by Ortega and colleagues identified the differences between winning and losing teams in 58 Six Nations games [75]. They found that winning teams scored more points and lost fewer set-pieces, compared to losing teams [75]. The practical applications for their findings were that ‘teams can use the information to set goals for players and teams in both practices and matches’ [75]. As most teams set themselves out to out-score the opposition, as well as win all of their set-pieces, the practical applications offered by the study offers very little applicable information to coaches. However, from a research perspective, the study has identified three areas for future studies to investigate; how teams score points, win line-outs and win scrums. A series of studies by Wheeler and colleagues [72, 76], analysed the skills that led to tackle breaks, an outcome identified as an effective means of scoring points in rugby union [72]. The key skills associated with tackle breaks were fending and evasive manoeuvres. Thus, the researchers suggested coaches develop evasive agility training programmes to improve their players’ ability. As these ‘how’ studies were able to investigate further into specific skills and events, the authors were able to provide more specific practical applications for those directly involved in rugby. To facilitate the transfer and adoption of research outcomes from research to practice, it is suggested that the practical application provided by video analysis research come from the findings of ‘how’ studies, and the results of ‘what’ studies inform the research questions of ‘how’ studies.

## Conclusions

The aim of this paper was to provide a critical review of video analysis research in rugby union. The review identified a large disparity in the type of data gathered in the studies and the level of statistical analysis conducted within the studies. The studies were categorised based on the outcome of the study (‘physical demands’, ‘performance’ or ‘injury related’) and the type of analysis (‘what’ or ‘how’) to facilitate more homogenous comparisons during the review process.

There was a large range in the sample sizes of the studies. The review raised concerns over the generalisability of the findings used in the majority of the studies reviewed and recommends that researchers adopt the practice of sample size calculations to ensure that studies are adequately powered.

Half of the studies appraised did not fully define the variables used in their analyses. There were also differing definitions of a variable between studies. These findings highlight the need for a consensus on the definitions of variables used in rugby union research so that the findings from different studies are more comparable (i.e. like the injury definitions for rugby union [77]).

Despite a common criticism that video analysis research has a tendency towards reductionism [8, 22, 27], all the ‘how’ studies reviewed included contextual variables in their analysis with 86% including more than two categories.

Finally, an aim of video analysis research is to provide information to coaches and practitioners to inform practice [26]. This information should be useful to a coach by not only answering the question of *what* happens in a match but also *how* it happens [77]. To assist in this process, it is suggested that researchers in this field start by developing research questions to identify the *what*, to provide novel findings used to develop the research questions to understand the *how*. This process will allow researchers to provide coaches with practical information, based on the results of *how* studies, which is useful and applicable to develop practice.

## Additional file

Additional file 1: Rugby union video analysis research database. (XLSX 73 kb)

## Abbreviation

KPIs: Key performance indicators
